# Material Models and Properties in the Finite Element Analysis of Knee Ligaments: A Literature Review

**DOI:** 10.3389/fbioe.2014.00054

**Published:** 2014-11-17

**Authors:** Fabio Galbusera, Maren Freutel, Lutz Dürselen, Marta D’Aiuto, Davide Croce, Tomaso Villa, Valerio Sansone, Bernardo Innocenti

**Affiliations:** ^1^IRCCS Istituto Ortopedico Galeazzi, Milan, Italy; ^2^Center of Musculoskeletal Research Ulm (ZMFU), Institute of Orthopedic Research and Biomechanics, Ulm University, Ulm, Germany; ^3^Department of Chemistry, Materials and Chemical Engineering, Politecnico di Milano, Milan, Italy; ^4^Department of Orthopaedic, Università degli Studi di Milano, Milan, Italy; ^5^BEAMS Department (Bio Electro and Mechanical Systems), École Polytechnique de Bruxelles, Université Libre de Bruxelles, Brussels, Belgium

**Keywords:** knee ligaments, finite element, material models, anisotropy, non-linearity, anterior cruciate ligament

## Abstract

Knee ligaments are elastic bands of soft tissue with a complex microstructure and biomechanics, which are critical to determine the kinematics as well as the stress bearing behavior of the knee joint. Their correct implementation in terms of material models and properties is therefore necessary in the development of finite element models of the knee, which has been performed for decades for the investigation of both its basic biomechanics and the development of replacement implants and repair strategies for degenerative and traumatic pathologies. Indeed, a wide range of element types and material models has been used to represent knee ligaments, ranging from elastic unidimensional elements to complex hyperelastic three-dimensional structures with anatomically realistic shapes. This paper systematically reviews literature studies, which described finite element models of the knee, and summarizes the approaches, which have been used to model the ligaments highlighting their strengths and weaknesses.

## Introduction

Numerical methods have been used for decades for the simulation of the biomechanical behavior of the knee joint. Starting from the earliest analytical or numerical models solved by in-house computer programs (Crowninshield et al., 1976; Wismans et al., 1980; Hefzy and Grood, 1983), the complexity of the kinematics and of the capacity of the knee joint to withstand high loads while allowing for a high mobility of the joint have always challenged and fascinated the scientific community. Modern finite element models are usually based on magnetic resonance imaging (MRI) and/or computed tomography (CT) scans and possess a high degree of anatomical realism (e.g., Pena et al., 2006; Kazemi and Li, 2014). Besides the simulation of the intact joint, models are widely used for the prediction of the effects of degenerative pathologies, traumatic events as well as surgical repair and replacement strategies (e.g., Yoon et al., 2010; Fitzpatrick et al., 2014; Innocenti et al., 2014).

Although the geometrical accuracy of the three-dimensional reconstruction, which can be achieved relatively easily, the development of an accurate finite element model of the knee joint is still a complex task. The ligaments of the knee are among the most complicated structures to simulate and at the same time most critical in determining the biomechanics of the joint. Indeed, ligaments have peculiar mechanical characteristics (described in detail below), which present technical challenges to researchers. Furthermore, valid and trusted values of the material properties obtained with experimental mechanical testing are needed in order to have a realistic response of the joint as predicted by the numerical models.

A wide range of different approaches to these challenges has been presented in the literature, and is the subject of the present review. After a summary of the main anatomical and biomechanical properties of the knee ligaments, we present a systematic review of the literature aimed to cover: (1) the type of elements, which have been used to simulate the knee ligaments; (2) the constitutive material laws employed in published studies; and (3) the original data sources, e.g., from *in vitro* mechanical testing of isolated ligaments, which have been used in the development and validation of the models.

## Functional Anatomy and Biomechanics of the Knee Ligaments

**Figure 1:**
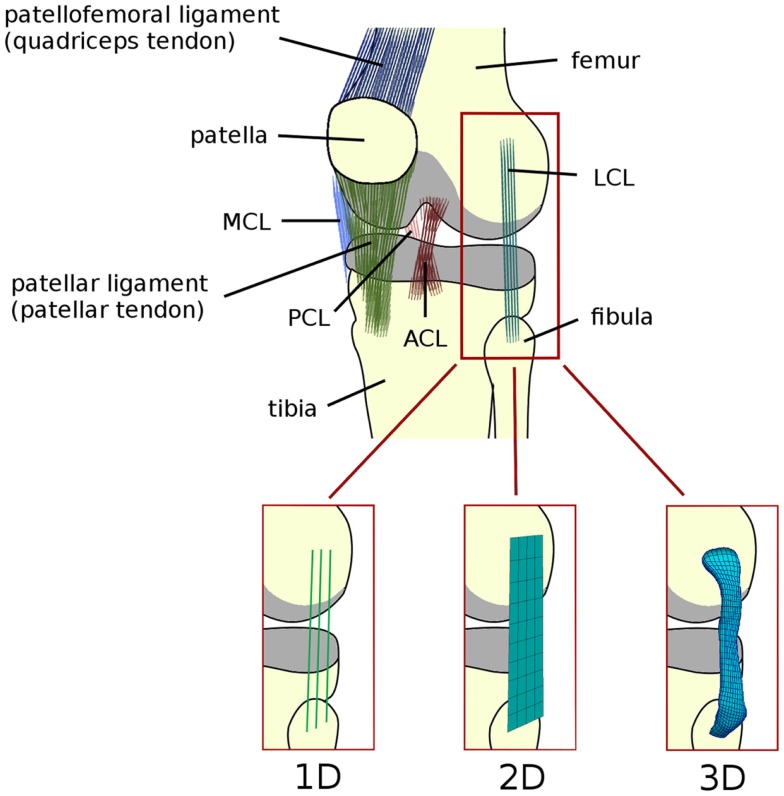
**Schematic representation of the anatomy of the knee joint, depicting the ligaments (ACL, anterior cruciate ligament; PCL, posterior cruciate ligament; MCL, medial collateral ligament; LCL, lateral collateral ligament)**. Articular cartilage is shown in gray. Alternative names commonly used for the patellofemoral and patellar ligaments are reported in brackets. Schematic representations of 1D (springs, trusses, and beams), 2D (shell and membrane), and 3D elements (solid) used to model the knee ligaments are shown.

Similar to other biological soft tissues, the ligaments of the knee (Figure 1) and other articular joints are constituted by a water-rich ground substance reinforced with collagen fibers (Daniel et al., 1990). The ground substance contains proteoglycans, which together with hyaluronic acid are able to attract water from the external environment, creating a kind of gel (Daniel et al., 1990). The capability of the matrix to capture water molecules and therefore to maintain a high water content even when stretched, results in the assumption of incompressibility of the tissue, which is incorporated in most constitutive models (Weiss and Gardiner, 2001; Weiss et al., 2005).

**Figure 2:**
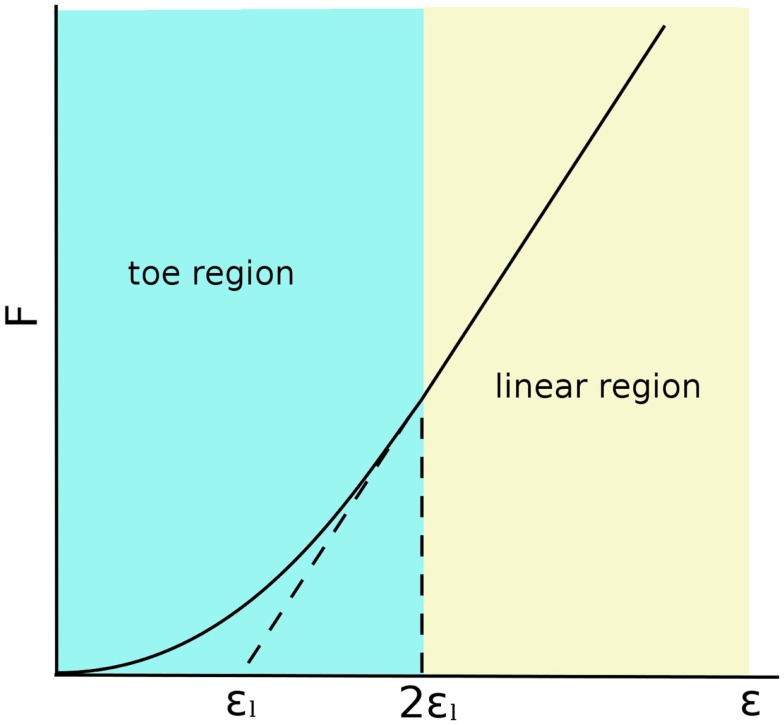
**Force–strain behavior of a generic ligament following the model described by Blankevoort et al. (1991a)**. 2ε_l_ is the threshold strain, which indicates the change from the toe to the linear regions.

The matrix also contains a moderate number of fibroblasts, which are responsible for synthesizing collagen molecules, and constitute the fibers providing the tensional stiffness and resistance to the tissue (Nimni, 1983). The collagen skeleton of ligaments is a hierarchical structure, which includes microfibrils organized in fibrils or fascicles. In the unloaded configurations, collagen fibrils are crimped, i.e., arranged in a helical or waveform pattern (Diamant et al., 1972; Comninou and Yannas, 1976). When the ligament is stretched, the crimping progressively disappears as the fibrils become aligned with the loading direction. This structure gives the ligament a characteristic force–elongation curve, which can be subdivided in two zones: (1) a toe region with low stiffness and non-linear response, in which the fibrils lose their crimping; (2) a higher stiffness region in which the curve is almost linear, which corresponds to the stretching of the collagen fibrils (Trent et al., 1976; Weiss and Gardiner, 2001) (Figure 2). This behavior is well represented by the constitutive models, which were developed for the numerical simulation of the biomechanics of the knee joint, which are described below.

## Review Methods

A Pubmed search with keywords “finite element” and “knee” was performed. The retrieved abstracts were then screened in order to determine, which papers were fulfilling the following inclusion criteria: (1) the paper describes a finite element model of the human lower limb, of the knee or of isolated knee ligaments; (2) the paper reports the material properties assigned to the knee ligaments or at least a reference to a literature source. Precedence was given to models of the entire knee joint and not on isolated ligaments; however, the latter papers were not excluded if retrieved by the aforementioned Pubmed search. Multibody models (i.e., based on rigid body dynamics) were not considered if not referenced by other finite element studies, for the sake of simplicity. When necessary, the full text of the retrieved papers was also screened. The reference lists of the papers were then analyzed in order to retrieve additional relevant papers, which had not been identified by the Pubmed search. The results of this systematic search were then subdivided into those showing a one-dimensional (1D) model of the knee ligaments, and those reporting two-dimensional (2D, surface elements), or three-dimensional (3D, solid elements) models.

**Figure 3:**
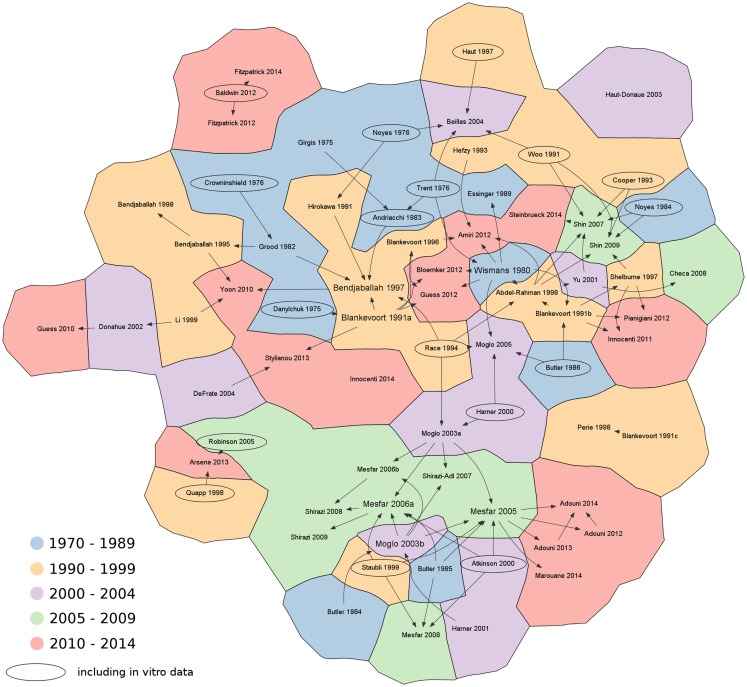
**Citation chart showing the sources of each paper retrieved in the literature using 1D elements to represent the knee ligaments**. Arrows indicate the papers used as reference for the material properties of the ligaments. Studies including *in vitro* data are highlighted with an oval border. For the sake of brevity, only the name of the first author is shown. References not reported in the main text: Abdel-Rahman and Hefzy, 1998; Adouni and Shirazi-Adl, 2013; Adouni and Shirazi-Adl, 2014; Adouni et al., 2012; Andriacchi et al., 1983; Arsene and Gabrys, 2013; Beillas et al., 2004; Blankevoort and Huiskes, 1996; Blankevoort et al., 1991c; Cooper et al., 1993; Danylchuk, 1975; DeFrate et al., 2004; Donahue et al., 2002; Essinger et al., 1989; Grood and Hefzy, 1982; Guess et al., 2010; Harner et al., 2000; Haut and Haut, 1997; Haut Donahue et al., 2003; Innocenti et al., 2011; Li et al., 1999; Marouane et al., 2014; Mesfar and Shirazi-Adl, 2006a; Mesfar and Shirazi-Adl, 2006b; Mesfar and Shirazi-Adl, 2008; Moglo and Shirazi-Adl, 2003a; Moglo and Shirazi-Adl, 2003b; Moglo and Shirazi-Adl, 2005; Noyes et al., 1984; Perie and Hobatho, 1998; Pianigiani et al., 2012; Robinson et al., 2005; Shelburne and Pandy, 1997; Shirazi and Shirazi-Adl, 2009; Shirazi et al., 2008; Shirazi-Adl and Mesfar, 2007; Stylianou et al., 2013.

**Figure 4:**
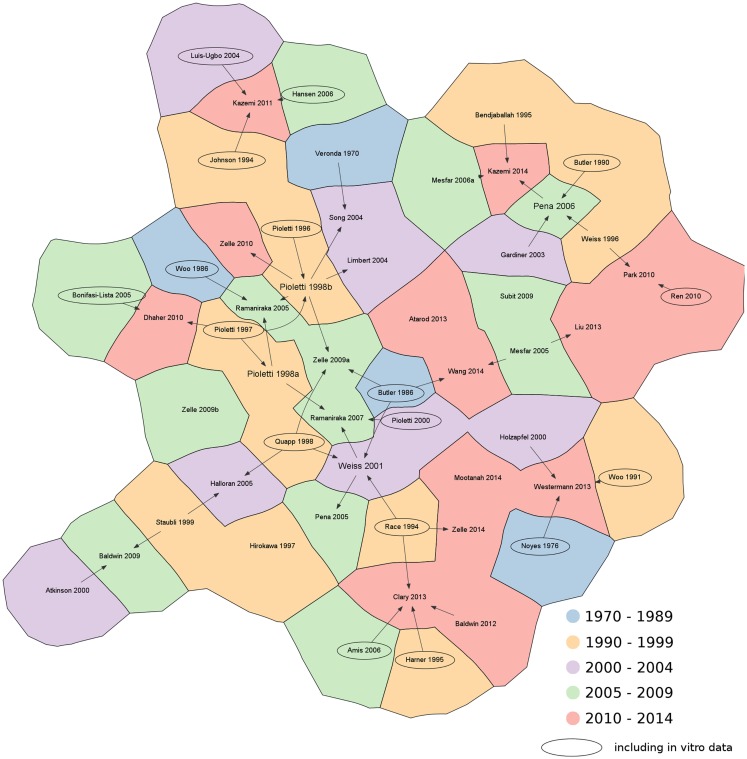
**Citation chart showing the sources of each paper retrieved in the literature using 2D and 3D elements to represent the knee ligaments**. Arrows indicate the papers used as reference for the material properties of the ligaments. Studies including *in vitro* data are highlighted with an oval border. For the sake of brevity, only the name of the first author is shown. References not reported in the main text: Amis et al., 2006; Atarod et al., 2013; Bonifasi-Lista et al., 2005; Butler et al., 1990; Dhaher et al., 2010; Hansen et al., 2006; Hirokawa and Tsuruno, 1997; Johnson et al., 1994; Louis-Ugbo et al., 2004; Mesfar and Shirazi-Adl, 2006a; Park et al., 2010; Pioletti et al., 1996; Ren et al., 2010; Subit et al., 2009; Wang et al., 2014.

Subsequently, the literature search was deepened in order to identify the original sources of the material properties, either based on assumptions, *in vitro* or *in vivo* measurements. Citation graphs tracking the origin of the material property values were created, specifically for the 1D and for the 2D–3D models (Figures 3 and 4).

## Results of the Literature Review

The first Pubmed search retrieved 650 items. After screening, 69 relevant papers were selected. The analysis of the references returned nine additional relevant papers. Several papers used values of the material properties retrieved from other modeling studies, and did not provide citations to the original data sources. Based on the detailed analysis of the literature, 18 papers reporting *in vitro* data about mechanical testing of the knee ligaments were identified as the original sources of the material properties. For the sake of clarity, the retrieved studies were subdivided in papers using 1D elements to represent the knee ligaments and those using elements with higher dimensionality (2D, 3D).

### 1D models

Line elements such as springs, trusses, and beams are frequently used to model the mechanical role of the ligaments in the knee joint (Figure 1). Bundles of line elements, which cover approximately the insertion areas of the ligaments are the most common solution (e.g., Bendjaballah et al., 1995), but single elements to describe each ligamental bundle were also used (Yu et al., 2001). The first mechanical tests conducted in the 70s (Girgis et al., 1975; Trent et al., 1976) showed a clear non-linearity of the force–elongation curves of all knee ligaments. Based on these observations, early numerical models of the knee joint used elements able to represent non-linear force–strain or force–elongation behaviors, e.g., quadratic (Wismans et al., 1980) or quadratic in the toe region and linear afterward (Blankevoort et al., 1991a). This behavior can be formulated as follows (Figure 2):

(1) $$\begin{array}{cc} f=\frac{1}{4}k\frac{\varepsilon^{2}}{\varepsilon_{l}}, & 0\leq \varepsilon \leq 2\varepsilon_{l}\\ f=k\left(\varepsilon -\varepsilon_{l}\right), & \varepsilon >2\varepsilon_{l}\\ f=0, & \varepsilon <0 \end{array}$$

where *f* is the axial force sustained by the ligament, *k* is a stiffness parameter, ε is the strain, and 2ε_l_ is the threshold strain, which indicates the change from the toe to the linear regions. In both cases, the elements representing the ligaments were able to sustain only tensile loads, and offered no resistance to compression or shear. In some papers (Yu et al., 2001; Checa et al., 2008), the force–strain behavior expressed in Eq. 1. has been modified as follows:

(2) $$\begin{array}{cc} f=k1{\left(L-L_{0}\right)}^{2}, & 0\leq \varepsilon \leq 2\varepsilon_{l}\\ f=k2\left(L-\left(1+\varepsilon_{l}\right)L_{0}\right), & \varepsilon >2\varepsilon_{l}\\ f=0, & \varepsilon <0 \end{array}$$

where *L* is the current length of the ligament, *L*_0_ is its slack length, and *k*1 and *k*2 are two stiffness parameters describing the toe and the linear regions, respectively. Linearized forms of Eqs 2 were also used (Shin et al., 2007):

(3) $$\begin{array}{cc} f=\frac{k_{l}}{2}\left(L-L_{0}\right), & 0\leq \varepsilon \leq 2\varepsilon_{l}\\ f=k_{l}\left(L-\left(1+\varepsilon_{l}\right)L_{0}\right), & \varepsilon >2\varepsilon_{l}\\ f=0, & \varepsilon <0 \end{array}$$

where *k_l_* is a linear stiffness parameter. Care should be taken in the comparison of the stiffness parameters *k*, *k*1, and *k*2 and *k*_l_ since they are expressed in different units.

These formulations are still widely used to model the ligaments in 1D (Table 1). It should be noted that the values of the material properties listed in Table 1 show considerable variability, especially regarding the reference strain. In some cases, the authors only stated the use of non-linear elements without any additional information on the constitutive equations (Shin et al., 2009; Yoon et al., 2010). In contrast, the use of linear 1D elements appears to be very limited in simulation studies (Shin et al., 2007; Innocenti et al., 2014; Steinbruck et al., 2014).

**Table 1:** **Material properties used to model the non-linear behavior of the ACL and PCL with non-linear 1D elements**.

ACL			PCL			Eq.	Reference
Stiffness	ε_L_	ε_0_	Stiffness	ε_L_	ε_0_	
aAC: *k* = 5000 N	0.03	aAC: 0.06	aPC: 9000 N	0.03	aPC: 0.24	(1)	Blankevoort et al. (1991a), Bloemker et al. (2012), Guess and Stylianou (2012)
pAC: *k* = 5000 N		pAC: 0.10	pPC: 9000 N		pPC: 0.03	
*k* = 5000 N	0.03	aAC: 0.16	*k* = 9000 N	0.03	aPC: −0.068	(1)	Amiri and Wilson (2012)
		mAC: 0.10			mPC: −0.169	
		pAC: 0.10			pPC: −0.169	
Toe region:	0.03	aAC: 0	Toe region:	0.03	aPC: 0.004	(2)	Yu et al. (2001), Checa et al. (2008)
aAC: *k*_1_ = 22.48 N/mm^2^		pAC: 0.051	aAC: *k*_1_ = 31.26 N/mm^2^		pPC: 0.05	
pAC: *k*_1_ = 26.27 N/mm^2^			pAC: *k*_1_ = 19.29 N/mm^2^			
Linear region:			Linear region:			
aAC: *k*_2_ = 83.15 N/mm^2^			aAC: *k*_2_ = 125 N/mm^2^			
pAC: *k*_2_ = 83.15 N/mm^2^			pAC: *k*_2_ = 60 N/mm^2^			
aAC: *k*_l_ = 108 N/mm	0.03	aAC: 0.02	aAC: *k*_l_ = 125 N/mm	0.03	aPC: −0.10	(3)	Shin et al. (2007), Steinbruck et al. (2014)
pAC: *k*_l_ = 108 N/mm		pAC: 0.02	pAC: *k*_l_ = 60 N/mm		pPC: −0.02	

*“Stiffness” indicates the stiffness parameter used in the relevant equations; “Eq.” indicates which force–strain equations were used, as described in the paper (Eqs 1–3). Other abbreviations: aAC, anterior bundle of the ACL; mAC, middle bundle; pAC, posterior bundle; aPC, anterior bundle of the PCL; mPC, middle bundle; pPC, posterior bundle; ε_L_, threshold strain, see Eqs 1 and 2; ε_0_, reference strain*.

Knee extension is usually considered as the reference state from which different motions can be simulated. In this state, the ligaments are strained and therefore already sustaining a tensile load (Daniel et al., 1990). Reference strains, also called pre-strains, are difficult to estimate experimentally, and assumptions were often made in order to circumvent this problem and to correctly simulate the initial state of knee extension (Wismans et al., 1980). Blankevoort et al. (1991a) used an iterative approach in order to minimize the differences between the predicted and experimentally measured flexion motions by altering the values of the reference strains of the ligaments. These data were used as a reference in a whole series of papers published by the École Polytechnique de Montréal (e.g., Mesfar and Shirazi-Adl, 2005). Another approach based on optimization to fit *in vitro* results was used by Baldwin et al. (2012) and served as basis for subsequent papers by the same research group (Fitzpatrick et al., 2012, 2013, 2014). Experimental measurements and sensitivity analyses about the reference strains were also performed (Bertozzi et al., 2007; Bloemker et al., 2012).

The ligaments of the knee do not only exert forces on the insertion areas in the direction connecting the insertion and origin, but exhibit wrapping behavior between themselves [anterior cruciate ligament (ACL) and posterior cruciate ligament (PCL)] or with bones [medial collateral ligament (MCL) with tibia and MCL with femur]. This behavior limits the accuracy of simple line elements to model these three ligaments if special techniques to simulate wrapping are not employed. Based on the analytical model proposed by Hefzy and Grood (1983), Blankevoort and Huiskes (1991b) first integrated the simulation of the MCL–tibia wrapping in a finite element model. The tibial surface was modeled as a curve in space upon which a moving contact point with the MCL was defined. The MCL was then divided into two line elements passing through the contact point. Similar approaches were used afterward (Bendjaballah et al., 1997, 1998), but the vast majority of papers employing 1D elements to model the ligaments neglected this phenomenon. In some specific cases in which wrapping markedly changes the force distribution and direction (e.g., when the valgus laxity is of interest), this limitation may significantly limit the accuracy of the results. However, the general effect of neglecting ligament wrapping was estimated to be not dramatic in other cases (Blankevoort and Huiskes, 1991b).

During the literature search and review, 14 *in vitro* papers were identified as reference for the material properties of the knee ligaments (Figure 3). The series of papers from the group of Butler and coworkers (Noyes and Grood, 1976; Butler et al., 1984, 1985, 1986) and the more recent paper by Race and Amis (1994) are worthy of note. Experimental data about the cruciate and collateral ligaments were also published by another research group (Woo et al., 1986, 1991; Harner et al., 1995, 2001). Experimental sources often cited were also Atkinson et al. (2000) and Staubli et al. (1999) regarding the patellar and patellofemoral ligaments.

It should be noted that in many finite element papers, the values of the material properties were retrieved from previous numerical studies, without direct references to experimental studies. A preference toward source papers, which explicitly showed values of the stiffness parameters and reference strain of the ligaments (such as Wismans et al., 1980 and Blankevoort et al., 1991a), is clearly seen in Figure 3. As a matter of fact, these studies refer to a small set of old experimental tests, which were shown already by Blankevoort et al. (1991a) to be rather inconsistent or inaccurate. Besides, values of the material properties used in previous models showed considerable variability (Table 1), thus making further questionable the model predictions and the comparability of data obtained with different models. We therefore recommend that authors of numerical models of the knee concentrating on the ligaments should not only focus on a single source of data, but perform a wider literature search as well and take into consideration also newer experimental datasets such as e.g., those reported by Race and Amis (1994) and Harner et al. (1995). It should be noted that the use of consolidated and valid sources for the material properties does not eliminate the need for a proper model validation.

### 2D–3D models

The most intuitive way to include the ligaments in a 3D model of the knee is by using solid elements (Figure 1). Indeed, MRI scans in combination with 3D reconstruction software offer an accessible way to create detailed models of the joint including the geometry of the ligaments as well as their insertion sites for the specific patient. MRI scans can also be easily combined with CT, which offers high resolution imaging of the bony structures, by means of registration software. This approach also facilitates the simulation of ligament wrapping by the use of surface-to-surface contact, which is implemented in most free and commercial finite element packages. The accuracy of the simulation of stresses that are not purely tensional, such as those arising due to contact with bone and those close to the insertion areas, is also improved (Weiss and Gardiner, 2001).

However, ligaments have a peculiar mechanical behavior, being strongly anisotropic and not able to sustain compression, which makes their simulation with solid elements less attractive if compared to line elements. The pre-strain in the reference state is also more challenging to be simulated using solid elements. One approach in between, which combines the ease of implementation of the 1D elements and the anatomical realism of the 3D elements, is to embed springs or trusses in a 3D matrix having a simple constitutive law, such as linear isotropic elasticity or neo-Hooke hyperelasticity. This approach was used in some previous studies, especially employing 2D (surface) elements such as shells or membranes reinforced with non-linear line elements to accomplish the anisotropy of the ligaments (Halloran et al., 2005; Baldwin et al., 2009; Zelle et al., 2009a,b, 2010, 2014; Clary et al., 2013).

Simple continuum material models that do not take into account the anisotropy of the ligamentous tissue, namely hyperelastic neo-Hooke (Mootanah et al., 2014) or Mooney–Rivlin materials (Liu and Zhang, 2013) were also used. However, anisotropic hyperelastic continuum models are more often employed. Only one paper (Westermann et al., 2013) employed an anisotropic hyperelastic model directly available in a finite element package, the Holzapfel–Gasser–Ogden model (Holzapfel et al., 2000; Gasser et al., 2006) implemented in ABAQUS (Simulia, Providence, RI, USA).

Constitutive models were also purposely developed to model biological soft tissues. The Veronda–Westmann model published in 1970 (Veronda and Westmann, 1970) provides an exponential stress–strain relationship and is conveniently available in open-source finite element software FEBio (Maas et al., 2012), but is limited to the isotropic behavior in its conventional formulation. This model was implemented in MARC (MSC Software, Newport Beach, CA, USA) for the simulation of the ACL (Song et al., 2004).

Pioletti and coworkers (Pioletti et al., 1998a,b; Pioletti and Rakotomanana, 2000) developed a hyperelastic, incompressible, and viscoelastic isotropic law, which was used in following works of the group (Ramaniraka et al., 2005, 2007). In this formulation, the elastic part is based on the isotropic Veronda–Westmann material law and the novelty is mainly constituted by the viscous behavior. However, in two papers (Ramaniraka et al., 2007 and Ramaniraka et al., 2005) this constitutive law was used to perform static analyses, and therefore did not exploit its viscoelastic capabilities.

A model which was widely used in the following publications was presented by Weiss et al. (1996), and describes an incompressible, fiber-reinforced material able to simulate large displacements. This material model was designed to closely fit the response of the biological soft tissues, especially ligaments, based on experimental data (Quapp and Weiss, 1998). Within this model, pre-strain was also simulated by the same group (Gardiner and Weiss, 2003) by performing an initial unloaded step to search for the reference configuration. The material model is available in FEBio in two forms, in which the matrix is either a Mooney–Rivlin or a Veronda–Westmann material. Peña and coworkers implemented the same model in ABAQUS by means of a Fortran user subroutine (UMAT) (Pena et al., 2005, 2006). A similar material formulation expanded to incorporate fluid flow (poroelasticity) and viscoelasticity was implemented in ABAQUS by Kazemi and coworkers (Kazemi et al., 2011; Kazemi and Li, 2014) by means of a UMAT and was used to investigate the creep behavior of the knee joint. Limbert et al. (2004) focused their attention to the possibility of simulating pre-strain in a ligament, and developed an anisotropic, fiber-reinforced incompressible hyperelastic material formulation in which an additional load step to calculate the reference configuration due to pre-strain is not necessary. To our knowledge, this material model has not yet been employed in other studies.

The analysis of the data sources used for the choice of the values of the material properties for 3D models showed similar results to those for the 1D models (Figure 4). Experimental sources (e.g. Butler et al., 1986; Woo et al., 1991; Race and Amis, 1994; Quapp and Weiss, 1998) were widely cited. The research group of Pioletti and coworkers (Pioletti, 1997; Pioletti and Rakotomanana, 2000) presented additional *in vitro* results, which have been widely used not only by the same group, but also in other papers (Limbert et al., 2004; Zelle et al., 2009a, 2010) to provide experimental evidence. With respect to previous data, these experimental tests also provided time-dependent results obtained at different strain rates, which would be useful in the implementation of dynamic models of the knee. In contrast to the 1D models, key references cited by a high number of papers (such as Wismans et al., 1980; Blankevoort et al., 1991a) could not be identified.

## Choice of Appropriate Elements and Material Models

The approaches used to simulate the mechanical behavior and role of the ligaments of the knee range from very simple (e.g. linear beams and springs) to complex models (e.g., anisotropic hyperelastic 3D materials, or biphasic formulations). The user planning to develop a computational model of the knee has therefore a wide range of possible choices, which should be evaluated according to the research questions and the desired applications of the model.

Weiss and Gardiner (2001) distinguished between microstructural models, which represent the mechanical role of each component of the ligamentous tissue, and phenomenological models, which simulate the global material behavior without directly referring to the tissue composition, therefore without a direct physical interpretation. The latter models are the focus of the present paper, and are the solution of choice for most models aimed to study the knee kinematics and load bearing, total or partial knee replacements, meniscus, and even ligament reconstruction and grafting.

Line elements have the distinct advantages of an easy implementation, a low computational cost and the possibility to exactly replicate non-linear force–elongation curves from experimental tests as well as pre-strain. Viscoelastic behavior can be also easily modeled, if required. As mentioned above, disadvantages include the lack of information about the behavior in the transverse plane and the need for special techniques to simulate ligament wrapping. Nevertheless, non-linear 1D elements are still a recommended choice and a commonly used method for the simulation of the global behavior of the knee and for total as well as unicompartmental knee replacements.

Solid elements offer a better anatomical realism and overcome the limitations in the simulation of wrapping. Besides, they have the potential to predict quantities not accessible with the used of 1D elements such as local strains and accurate load transfer between ligaments and surrounding tissues, which may be of interest for specific applications. However, their inherent higher complexity may lead to high computational costs, difficulties in the validation and evaluation of the results, and in some cases to the use of oversimplified material constitutive models, which should be avoided. We recommend their use only when the focus of the study is the biomechanics of the ligament itself, its interaction with the surrounding tissues or other complex research questions in which a correct 3D representation of the behavior of the ligament is required in order to obtain an accurate answer. It should be noted that, depending on the specific application, other anatomical structures such as menisci and articular cartilage may have a high significance in the determination of the results, and should therefore be modeled and validated properly.

## Verification and Validation

Two key aspects for the development of numerical models of articular joints such as the knee are the verification and the validation. Even if these terms are sometimes used with similar meanings, as effectively synthesized by Roarche (1998) and Viceconti et al. (2005), verification is about solving the equations right, and validation is about solving the right equations. For the specific case of biomechanical simulation of the knee joint with commercial finite element software, verification is usually limited to the choice of an appropriate mesh density, the plausibility analysis of the solution of the contact problems and the check of the convergence of the iterative solution.

Validation is mostly performed by comparison of the results with controlled *in vitro* experiments. Experimental set-ups like the Oxford rig (Zavatsky, 1997) or the Kansas Knee Simulator (Maletsky and Hillberry, 2005) allow for the application of physiological loads and motions, and for the measurement of the resulting kinematics, which can then be compared with the numerical predictions. However, these experimental methods do not offer easy access to local variables such as strains in the ligaments, and therefore allow only for a partial validation of the numerical models. The use of sophisticated sensors such as differential variable reluctance transducers (DVRTs) (e.g., in Withrow et al., 2006) or optical strain measurements (e.g., in Freutel et al., 2014) offer a partial solution to this limitation, at the price of a higher level of complexity of the experimental set-up.

An alternative, indirect way to perform validation is by comparison with available data, such as data from literature or from previous experiments (Henninger et al., 2010). In this case, the user has no direct control on the experiments and on their quality and degree of variability. For this reason, validation against purposely performed experiments is generally preferred to indirect validation (Henninger et al., 2010).

Similarly to the choice of the most appropriate constitutive model described above, the design of a correct validation procedure is mainly based on the scope of the desired applications and on the output variables of interest (Viceconti et al., 2005; Henninger et al., 2010). For example, if the aim of the study is the evaluation of the kinematics of a total knee replacement, a comparison of the predictions with experimental kinematical data may be sufficient. However, a model validated in such a way would not be adequate for the evaluation of local strains in the MCL, which would require a model specifically validated for the mechanical behavior of the single ligament.

## Future Developments

A number of topics concerning the biomechanics of the knee ligaments remain poorly understood. Fluid flow during deformation of the ligaments is one of these. Following the studies of the transient mechanics and mechanobiology of the intervertebral disk (e.g., Schroeder et al., 2006), models including osmotic swelling due to the presence of proteoglycans would allow for a more accurate prediction of their time-dependent response and the overcoming of the incompressibility assumption. However, experimental data about permeability for the ligamentous tissue is actually lacking (Weiss et al., 2005).

Another aspect which is poorly investigated is the simulation of ligament failure and injury. With current commercial software, anatomically realistic 3D models able to accurately simulate local strains could be easily enriched in order to predict crack initiation and propagation by the use of extended finite element methods (XFEM) (Moës et al., 1999). However, data available about ligament failure only concern uniaxial tension along the main axis of the ligament, and are therefore not sufficient for the development of adequate failure criteria to be implemented with XFEM (Weiss et al., 2005).

## Conclusion

The use of numerical models for the biomechanical simulation of the knee joint in healthy and pathological conditions and following repair surgeries or implantation of prostheses appears to be wide and consolidated. The ligaments, especially ACL and PCL, have been the focus of studies from the earliest models onward (e.g., Wismans et al., 1980) due to their high biomechanical importance in both knee kinematics and load bearing. The increase in computational power of modern computers and the wide availability of advanced non-linear finite element packages, including software free of charge for academic use (e.g., FEBio) will likely broaden the use of 3D elements for the ligaments with respect to 1D solutions. We recommend the authors to consider all the unique aspects of the biomechanics of the ligaments, such as non-linearity, anisotropy, pre-strain, and wrapping, and to select the most appropriate modeling approach based on the specific application, especially when 3D elements are used.

## Conflict of Interest Statement

The authors declare that the research was conducted in the absence of any commercial or financial relationships that could be construed as a potential conflict of interest.
